# Overweight according to geographical origin and time spent in France: a cross sectional study in the Paris metropolitan area

**DOI:** 10.1186/1471-2458-12-937

**Published:** 2012-10-31

**Authors:** Judith Martin-Fernandez, Francesca Grillo, Christine Tichit, Isabelle Parizot, Pierre Chauvin

**Affiliations:** 1 INSERM, U707, Research team on the social determinants of health and healthcare, 27 rue de Chaligny, Paris, 75012, France; 2 University Pierre et Marie Curie-Paris 6, UMR-S 707, Paris, France; 3 INRA, U1303, Research team on diet and social science, 65 boulevard de Brandebourg, Ivry sur Seine FRANCE, 94205, France; 4 CNRS, ERIS, Research team an social inequality, 48 boulevard Jourdan, Paris, 75014, France; 5 AP-HP, Saint Antoine hospital, Public health unit, Paris, France

## Abstract

**Background:**

For the first time in France in a population-based survey, this study sought to investigate the potential impact of migration origin and the proportion of lifetime spent in mainland France on body mass index (BMI) and overweight in adults living in the Paris metropolitan area.

**Methods:**

A representative, population-based, random sample of the adult, French speaking population of the Paris metropolitan area was interviewed in 2005. Self-reported BMI (BMI = weight/height^2^) and overweight (BMI ≥ 25) were our 2 outcomes of interest. Two variables were constructed to estimate individuals’ migration origin: parental nationality and the proportion of lifetime spent in mainland France, as declared by the participants. We performed multilevel regression models among different gender and age groups, adjusted for demographics and socioeconomic status.

**Results:**

In women, a parental origin in the Middle East or North Africa (MENA) was associated with a higher risk of being overweight (especially before the age of 55) and a higher BMI (between 35 and 54 years of age), and so were women of Sub-Sahara African parental origin in the middle age category. Only in the youngest men (< 35 years of age) did we observe any association with parental nationality, with a higher BMI when having a MENA parentage. Regarding the association between the proportion of lifetime spent in France and overweight, we observed that, in women, a proportion of 50% to 99% appeared to be associated with overweight, especially after the age of 35. In men, having spent more than half of one’s lifetime in France was associated with a higher risk of overweight among oldest men.

**Conclusions:**

Our results plea for potential cultural determinants of overweight in the migrant and migrants-born populations in the French context of the capital region. Taking into account the people’ family and personal migration histories may be an important issue in public health research and policies on overweight and obesity prevention.

## Background

The prevalence of overweight and obesity is rapidly increasing in many countries [1] and is posing a public health risk. Worldwide in 2008, according to WHO, 1.5 billion adults (age > 20) were overweight (BMI ≥ 25), of these over 200 million men and nearly 300 million women were obese (BMI ≥ 30). In France, in 2009, 46,4% were overweight and 14.5% of the adult population (18 years and older) was obese [2], while in 1997, these prevalence were respectively 38,3% and 8.5%. Overweight is an increasingly serious public health problem, because of its impact on health [3] and its important spread.

Ethnic variations in obesity and overweight have been observed in several studies [4-8]. In European countries, studies comparing overweight and obesity prevalence in ethnic groups are scarce [7,9] and even more in France. While, it would seem important to study and take ethnicity into account in France, one of the European countries with high immigration flows from the beginning of the 20^th^ century to the 1970s and that have continued since then, although they have recently been more limited. Indeed, because of this history, there are a large number of people of foreign origin in this country who are mainly concentrated in urban areas, especially the Paris metropolitan area (we estimated that people with at least one foreign parent account for 17.7% of the total population). Moreover, although scarce, some social and economic surveys indicate that the socioeconomic status of French citizens born to foreign parents is, overall, lower than that of French citizens born to French parents, even if the vast majority of them were born and raised in France [10]. Their level of education is lower, and, for a given level of education, their unemployment rate, job qualifications and income are notably lower as well [11].

Nevertheless in France, epidemiological data on immigrant populations are limited because characterizing ethnicity for routine purposes is illegal and is generally perceived as irrelevant for demographic, historical and sociopolitical purposes [12]. Citizenship or immigration status are the proxies usually used to identify geographical origins but they don’t seem to be relevant for identifying the complexity of ethnics origins and cultural education and lifestyle associated with it. At least two previous French studies attempted to collect additional information on migration profiles [13] and migration biographies [10] in an effort to link them with health indicators [14], but no study has ever done so for overweight. Based on the hypothesis that the nationality or nationalities of a person’s parents and the proportion of his/her lifetime spent in France can be meaningful indicators and proxies for his/her geographical origin, culture and lifestyle exposure, this study sought to examine the potential impact of migration origin and the proportion of lifetime spent in mainland France on overweight in adults living in the Paris metropolitan area, after adjusting for several other sociodemographic and socioeconomic characteristics.

## Methods

### Study population

The target population comprised adults (≥ 18 years old) leaving in Paris metropolitan area (Paris and its suburban *départements*, a region with a population of 6.5 million). Analyses are based on the first wave of the SIRS survey (a French acronym for Health, Inequalities and Social Ruptures) conducted between September and December 2005 among a representative sample of the adult French-speaking population in the Paris metropolitan area. This survey constituted the first wave of a socio-epidemiological population-based cohort study, which is a collaborative research project between the French National Institute for Health and Medical Research (INSERM) and the National Centre for Scientific Research (CNRS). This cohort study was approved by the French privacy and personal data protection authority (*Commission Nationale de l’Informatique et des Libertés*).

In the present paper, data collected in 2005 were examined cross-sectionally. The SIRS survey employed a stratified, multistage cluster sampling procedure. The primary sampling units were census blocks called “IRISs” (a French acronym for blocks for incorporating statistical information). They constitute the smallest census unit areas in France (with about 2000 inhabitants each in the Paris metropolitan area) whose aggregate data can be used on a routine basis. In all, 50 IRISs were randomly selected (over-representing the poorer neighborhoods) from the 2595 eligible IRISs in Paris and its suburbs. Subsequently, within each selected IRIS, households were randomly chosen from a complete list of dwellings in order to include at least 60 households in each surveyed IRIS. Lastly, one adult was randomly selected from each household by the birthday method. A questionnaire containing numerous social and health-related questions was administered face-to-face during home visits. Since the methodology used in this study has been explained in more detail elsewhere [15-19], we will only remind here that 29% of the people contacted declined to answer, and 5% were excluded because they did not speak French (3%) or because they were too sick to answer our questions (2%). For our analysis, we further excluded 23 respondents because their weight and height data were missing, so the final sample consisted in 3000 persons.

### Outcome

We used the Body Mass Index (BMI = weight/height^2^) as a proxy of overall fat mass and followed the usual cut-off point [20]. BMI was assessed using self-reported weight and height. BMI was analyzed in two distinct ways. First, it was considered as a continuous variable in multilevel linear regression models and, second, individuals defined as overweight in multilevel regression analysis were those with a BMI ≥ 25 at the time of the survey (all the individuals with normal weight - BMI < 25 - constituting the comparison group).

### Variables of interest

Two variables were constructed for our purposes: parental nationality and proportion of lifetime spent in France. Parental nationality combined both nationalities of every respondent’s parents. These combined nationalities were sorted by geographical areas. The reference group consisted of individuals born to two French parents (Tables 1 and 2). We then distinguished those born to two Middle Eastern or North African parents (MENA), two Western European or North American (WENA) parents, two sub-Saharan African (SSA) parents, two Asian parents, two South American parents, and two East European parents. Lastly, when parents were from different geographical areas, two categories were constructed: “one French and one foreign parent” - in which the main geographical origin of the foreign parent was Western Europe or North America (n = 46), the Middle East or North Africa (n = 27), or the sub-Sahara (n = 12) - and “mixed foreigners” (n = 11), which contained individuals born to non-French parents of different origins. For the purposes of our analysis, to compare groups of sufficient sizes, a global “other” category was created that included “mixed foreigners” and parental nationality groups with a very small sample size (South America or Eastern Europe).

**Table 1:** Characteristics of the surveyed population, Paris metropolitan area, France, 2005

		**Men**	**Women**	
		Mean (sd)*	Mean (sd)*	
**Age**		44.1(0.8)	46.2 (0.9)	
**Body Mass Index**		24.8 (0.1)	23.8(0.1)	
		%*	%*	***p***
**BMI class**				< 0.0001
	< 18.5	1.3%	7.3	
	[18.50-25.00]	57.5%	60.0%	
	[25.00-30.00]	33.5%	23.0%	
	[30.00-35.00]	5.5%	7.5%	
	≥ 35	2.2%	2.2%	
**Monthly household income (quartile, in € per CU**^**Ɨ**^**)**				0.19
	> 2362	26.5%	23.6%	
	1600-2362	24.6%	23.6%	
	[1059-1600]	25.2%	26.7%	
	< 1059	23.6%	26.1%	
**Education level**				0.15
	Tertiary	52.5%	50.3%	
	None or primary	8.6%	10.9%	
**Parental nationality**				0.0093
	Both French	72.5%	68.4%	
	Sub-Saharan African	4.3%	5.7%	
	Middle Eatern/ North African	9.6%	8.4%	
	Asian	2.6%	2.3%	
	Western European/ North American	5.5%	6.2%	
	One French and one foreign parent	3.7%	4.6%	
	Other	1.9%	4.5%	
**Proportion of life time spent in France**				0.024
	Less than 50%	13.0%	15.1%	
	50% to 99%	25.0%	20.2%	
	100%	62.0%	64.8%	
**Socio-occupational groups**				*<0.0001*
	Executives, managers	32.5%	20.8%	
	Intermediate occupations	18.5%	25.2%	
	Tradespeople. shop and market salespeople	6.6%	3.0%	
	Employees	13.2%	33.6%	
	Workers	22.1%	4.2%	
	Never employed	7.2%	13.3%	
**Having had children**				0.0001
	Yes	61.6%	69.8%	
	No	38.4%	30.2%	

*Weighted means and proportions.

^Ɨ^Consumption units.

**Table 2:** Overweight (BMI ≥ 25 kg/m2) prevalence and factors associated with overweight in univariate analysis, Paris metropolitan area, France, 2005

		**Men (n = 1177)**					**Women (n = 1823)**				
		**n**	**overweight prevalence**	**raw OR**	**95%CI**	***p***	**n**	**overweight prevalence**	**raw OR**	**95%CI**	***p***
**Age-group**											
	18-34	343	26.1%	-	-	< 0.0001	526	21.9%	-	-	< 0.0001
	35-54	443	45.2%	2.22	[1.63-3.01]		680	33.4%	1.75	[1.35-2.28]	
	55 and over	391	56.2%	3.65	[2.66-5.02]		617	43.6%	3.23	[2.46-4.25]	
**Monthly household income (quartile, in € per CU**^**Ɨ**^**)**											
	> 2362	293	45.7%	-	-	0.4049	360	19.5%	-	-	< 0.0001
	1600-2362	260	39.4%	0.82	[0.58-1.15]		397	29.2%	1.52	[1.09-2.12]	
	[1059-1600[	317	38.6%	0.78	[0.56-1.09]		520	38.6%	2.27	[1.65-3.12]	
	< 1059	307	40.9%	0.95	[0.68-1.33]		546	41.7%	2.40	[1.73-3.33]	
**Education level**											
	Tertiary	536	36.4%	-	-	0.0002	811	19.9%			< 0.0001
	Secondary	497	44.4%	1.40	[1.08-1.80]		786	42.5%	2.60	[2.08-3.27]	
	None or primary	147	56.1%	2.12	[1.45-3.09]		245	56.7%	6.12	[4.47-8.38]	
**Socio-occupational groups**											
	Executives. managers	339	41.7%	-	-	< 0.0001	338	22.0%	-	-	< 0.0001
	Never employed	70	7.0%	0.15	[0,07-0,35]		184	28.7%	1.93	[1,27-2,92]	
	Tradespeople. shop and market salespeople	76	51.1%	1.46	[0,88-2,42]		45	30.6%	2.08	[1,06-4,06]	
	Intermediate occupations	221	36.5%	1.01	[0,71-1,43]		447	28.9%	1.54	[1,10-2,16]	
	Employees	166	50.1%	1.39	[0,95-2,04]		719	41.6%	2.69	[1,95-3,72]	
	Workers	306	47.7%	1.34	[0,97-1,85]		108	50.7%	3.82	[2,36-6,17]	
**Having had children**											
	Yes	746	50.2%	-	-	< 0.0001	1320	38.7%	-	-	< 0.0001
	No	431	26.9%	0.40	[0.31-0.52]		503	18.7%	0.40	[0.31-0.51]	
**Parental nationality**											
	Both French	824	39.2%	-	-	0.01	1235	28.7%	-	-	< 0.0001
	Sub-Saharan African	63	27.7%	0.59	[0.34-1.04]		105	51.7%	1.96	[1.29-2.97]	
	Middle Eastern/North African	120	47.8%	1.26	[0.85-1.86]		176	51.4%	2.17	[1.56-3.03]	
	Asian	29	38.9%	1.30	[0.61-2.77]		40	23.2%	0.59	[0.27-1.26]	
	Western European/North American	69	55.5%	1.80	[1.09-2.97]		100	33.5%	1.18	[0.76-1.82]	
	One French and one foreign parent	49	56.4%	2.01	[1.11-3.64]		88	36.9%	1.21	[0.76-1.91]	
	Other	21	46.8%	1.52	[0.63-3.67]		76	33.5%	1.32	[0.81-2.14]	
**Proportion of life time spent in France**											
	Less than 50%	169	27.4%	-	-	< 0.0001	269	34.1%	-	-	0.0006
	50% to 99%	315	51.2%	2.43	[1.63-3.61]		387	39.8%	1.37	[0.99-1.90]	
	100%	691	40.1%	1.49	[1.04-2.15]		1162	30.0%	0.85	[0.64-1.13]	

*weighted.

ƗConsumption units.

For each individual in the survey population, the proportion of his/her lifetime spent in mainland France was calculated as the ratio of the time lived in mainland France (hereinafter referred to as “France”) – i.e. the answer to the question: “How many years of your life have you lived in mainland France?” - over the respondent’s age. These proportions were divided into three categories: having spent “less than half of his/her life”, “between half and 99% of his/her life” and “his/her entire life” in France (< 50%, [50%-99%], and 100%, respectively).

### Adjustment variables

Six different characteristics were taken into account as adjustment variables. The first three concern demographics: gender, age (grouped into three categories: 18–34, 35–54, 55 and older) and the fact of having had children or not. Three other characteristics concern the individuals’ socioeconomic status (SES): education level (tertiary, secondary, none or primary), declarative monthly household income (calculated in euros per consummation unit and categorized into quartiles) and socio-occupational group (based on the classification of the French National Bureau of Statistics, which distinguishes between workers, employees, tradespeople/shopkeepers, intermediate occupations, executives/managers, and never having been employed, with retired or unemployed people classified according to their last job).

### Statistical analysis

All the means and proportions presented in this paper (except prevalence in Tables 3 and 4) were weighted to account for the complex sample design (not only the stratification that overrepresented the poorest neighborhoods, but also the fixed numbers of households selected by neighborhood and of adults interviewed by household) and for the post stratification adjustment for age and gender according to the general population census data. The design effect due to cluster sampling was also taken into account, using the “svy” command in Stata11 software. Means and proportions comparisons were tested by adjusted Wald tests and Pearson chi-squared tests respectively, with a significance threshold p < 0.05.

**Table 3:** **Factors associated with body mass index (BMI, in kg/m**^**2**^**) and overweight (BMI ≥ 25 kg/m**^**2**^**) in multivariate analysis by age group in women, Paris metropolitan area, France, 2005**

	**Women 18-34 years old (n = 522)**								**Women 35-54 years old (n = 679)**								**Women 55 years old and over (n = 614)**							
	**Body Mass Index**				**Body Mass Index ≥25 kg/m2**				**Body Mass Index**				**Body Mass Index ≥25 kg/m2**				**Body Mass Index**				**Body Mass Index ≥ 25 kg/m2**			
	**Mean BMI**	**Adjusted coef.**	**95% Cl**	***p***	OP*	**aOR**	**95% Cl**	***p***	**Mean BMI**	**Adjusted coef.**	**95% Cl**	***p***	OP*	**aOR.**	**95% Cl**	***p***	**Mean BMI**	**Adjusted coef.**	**95% Cl**	***p***	OP*	**aOR**	**95% Cl**	***p***
**Monthly household income (quartile, in € CU**^**Ɨ**^**)**																								
> 2362	21.29	-	Ref.	0.58	9.8	-	Ref.	0.70	22.82	-	Ref.	0.79	20.6	-	Ref.	0.79	23.51	-	Ref.	0.021	28.0	-	Ref.	0.016
1600-2362	22.50	0.77	[-0.53-2.07]		21.2	1.82	[0.66-5.01]		22.97	-0.47	[-1.55-0.61]		22.6	0.78	[0.40-1.49]		25.12	1.05	[-0.07-2.17]		44.9	1.71	[1.02-2.87]	
[1059-1600]	21.80	0.79	[-0.49-2.07]		23.2	1.76	[0.64-4.82]		24.32	-0.05	[1.18-1.08]		36.8	1.00	[0.52-1.92]		26.65	1.89	[0.69-3.09]		59.4	2.39	[1.38-4.14]	
< 1059	23.20	0.48	[-0.90-1.86]		29.6	1.74	[0.61-4.97]		25.56	-0.09	[-1.33-1.15]		48.9	0.92	[0.46-1.85]		26.69	1.57	[0.17-2.96]		53.1	1.54	[0.81-2.91]	
**Education level**																								
Tertiary	21.88	-	Ref.	0.0033	16.5	-	Ref.	0.03	22.73	-	Ref.	0.000	20.7	-	Ref.	0.0022	23.79	-	Ref.	0.017	30.2	-	Ref.	0.68
Secondary	23.92	1.54	[0.64-2.44]		34.0	2.02	[1.16-3.52]		24.82	1.39	[0.46-2.31]		41.5	1.97	[1.20-3.24]		25.54	0.97	[-0.08-2.03]		47.8	1.63	[1.00-2.65]	
None or primary	24.30	2.08	[-1.13-5.29]		42.9	3.65	[0.62-21.58]		27.44	3.28	[1.87-4.69]		65.7	3.52	[1.69-7.31]		27.15	1.90	[0.60-3.21]		62.1	2.36	[1.30-4.28]	
**Socio occupational groups**																								
Executives. managers	21.55	-	Ref.	0.81	11.0	-	Ref.	0.74	22.54	-	Ref.	0.74	17.9	-	Ref.	0.70	23.79	-	Ref.	0.57	31.7	-	Ref.	0.90
Never employed	22.34	0.02	[-1.38-1.43]		23.3	1.42	[0.50-4.02]		26.20	0.63	[-1.32-2.58]		61.8	2.05	[0.71-5.91]		25.51	-0.84	[-2.89-1.22]		50.0	0.77	[0.60-1.76]	
Tradespeople. shop and market sales people	22.49	0.61	[-0.58-1.81]		33.3	1.87	[0.76-4.61]		23.17	0.31	[-0.75-1.36]		29.4	1.30	[0.69-2.47]		24.76	0.15	[-1.00-1.30]		41.7	1.03	[0.60-1.76]	
Intermediate occupations	23.33	0.58	[-4.11-5.28]		22.0	2.22	[0.14-34.09]		23.42	0.21	[-1.97-2.39]		25.3	1.40	[0.42-4.64]		25.16	-0.57	[-2.76-1.62]		40.2	0.71	[0.26-1.94]	
Employees	23.57	0.36	[-0.97-1.68]		30.4	1.51	[0.57-3.97]		24.92	0.63	[-0.58-1.84]		41.7	1.49	[0.74-3.01]		26.35	0.49	[-0.79-1.76]		54.8	0.71	[0.62-1.99]	
Workers	23.03	-0.43	[-2.65-1.80]		25.0	0.98	[0.22-4.36]		26.44	1.47	[-0.30-3.24]		56.1	2.19	[0.84-5.73]		27.44	0.97	[-0.92-2.86]		59.6	1.17	[0.50-2.75]	
**Having had children**																								
Yes	23.72	-	Ref.	0.0005	32.0	-	Ref.	<0.01	24.46	-	Ref.	0.025	38.1	-	Ref.	0.012	25.69	-	Ref.	0.041	48.7	-	Ref.	0.040
No	21.82	-1.37	[-2.14-0.60]		16.3	0.47	[0.29-0.77]		22.59	-1.05	[1.96--1.13]		17.1	0.48	[0.27-0.85]		24.31	-0.99	[-1.95--0.04]		35.3	0.63	[0.40-0.98]	
**Parental nationality**																								
Both French	22.23	-	Ref.	0.090	19.6	19.6	-	Ref.	0.047	23.46	Ref.	0.011	27.3	-	Ref.	0.042	25.17	-	Ref.	0.71	43.4	-	Ref.	0.31
Sub-Saharan African	24.48	0.98	[-0.48-2.44]		35.4	1.01	[0.44-2.31]		26.80	2.00	[0.49-3.50]		62.8	3.07	[1.42-6.67]		28.51	0.97	[-3.02-4.96]		66.7	1.81	[0.28-11.84]	
Middle Eastern/ North African	24.14	1.14	[-0.04-2.32]		43.1	2.00	[1.02-3.91]		26.15	1.54	[0.40-2.68]		53.3	2.04	[1.14-3.65]		27.97	1.20	[-1.46-3.85]		76.2	4.31	[1.06-17.49]	
Asian	21.75	-1.26	[-2.14-1.57]		9.5	0.25	[0.05-1.22]		24.34	-0.19	[-2.59-2.22]		42.9	1.35	[0.41-4.50]		23.91	-1.71	[-5.92-2.51]		20.0	0.36	[0.49-3.04]	
Western European/ North American	22.69	-0.29	[-2.14-1.57]		25.0	0.92	[0.30-2.85]		23.68	-0.79	[-2.08-0.50]		32.7	0.81	[0.41-1.61]		25.78	-0.46	[-2.47-1.55]		52.0	1.22	[0.49-3.04]	
One French and one foreign parent	23.09	0.36	[-1.18-1.89]		24.1	0.98	[0.38-2.50]		24.66	0.97	[-0.72-2.66]		33.3	1.32	[0.54-3.21]		25.49	0.49	[-0.65-3.25]		61.5	1.98	[0.79-4.97]	
Other	22.09	-0.92	[-2.61-0.77]		13.8	0.37	[0.11-1.22]		24.52	0.79	[-1.26-2.85]		42.9	2.42	[0.82-7.11]		27.32	1.30	[-0.65-3.25]		61.5	1.98	[0.79-4.79]	
**Proportion of life time spent in France**																								
Less than 50%	23.20	-	Ref.	0.97	29.9	-	Ref.	0.89	24.98	-	Ref.	0.16	40.4	-	Ref.	0.011	26.66	-	Ref.	0.035	53.9	-	Ref.	0.32
50% to 99%	22.75	0.06	[-1.20-1.32]		22.1	0.83	[0.38-1.80]		25.09	0.75	[-0.31-1.81]		47.3	2.15	[1.22-3.76]		26.25	0.91	[-1.37-3.18]		50.4	2.08	[0.68-6.35]	
100%	22.58	0.13	[-0.92-1.19]		22.1	0.88	[0.47-1.67]		23.46	0.00	[-1.11-1.11]		27.0	1.30	[0.72-2.35]		25.08	-0.37	[-2.70-1.95]		44.1	1.64	[0.53-5.07]	

*Overweight prevalence (%).

ƗConsumption units.

**Table 4:** **Factors associated with body mass index (BMI, in kg/m**^**2**^**) and overweight (BMI ≥ 25 kg/m**^**2**^**) in multivariate analysis by age group in men, Paris metropolitan area, France, 2005**

	**Men 18-34 years old (n = 341)**								**Men 35-54 years old (n = 443)**								**Men 55 years old over (n = 390)**							
	**Body mass index**				**Body mass index ≥25 kg/m2**				**Body mass index**				**Body mass index ≥ 25 kg/m2**				**Body mass index**				**Body mass index ≥ 25 kg/m2**			
	**Mean BMI**	**Adjusted coef.**	**95% Cl**	***p***	OP*	**aOR**	**95% Cl**	***p***	**Mean BMI**	**Adjusted coef.**	**95% Cl**	***p***	OP*	**aOR**	**95% Cl**	***p***	**Mean BMI**	**Adjusted coef.**	**95% Cl**	***p***	OP*	**aOR**	**95% Cl**	***p***
**Monthly household income (quartile, in € per CU**^**Ɨ**^**)**																								
> 2362	22.56	-	Ref.	0.19	15.0	-	Ref.	0.54	25.15	-	Ref.	0.88	45.1	-	Ref.	0.96	26.00	-	Ref.	0.039	55.0	-	Ref.	0.25
1600-2362	23.60	0.91	[-0.44-2.26]		26.8	2.02	[0.63-6.46]		25.09	-0.42	[1.53-0.70]		42.6	0.86	[0.47-1.60]		26.42	-0.05	[-1.28-1.19]		57.1	0.87	[0.44-1.75]	
[1059-1600]	23.43	0.79	[-0.57-2.15]		28.1	1.99	[0.62-6.44]		25.28	-0.41	[-1.68-0.87]		46.6	0.99	[0.49-1.99]		25.36	-1.33	[-2.72-0.05]		52.5	0.54	[0.24-1.19]	
< 1059	23.91	1.55	[0.06-3.04]		30.0	2.56	[0.72-9.11]		25.14	-0.52	[-1.94-0.90]		44.4	0.96	[0.44-2.09]		26.88	0.40	[-1.06-1.86]		64.4	1.07	[0.45-2.54]	
**Education level**																								
Tertiary	23.17	-	Ref.	0.36	21.6	-	Ref.	0.86	24.89	-	Ref,	0.25	43.7	-	Ref.	0.97	25.63	-	Ref.	0.33	50.7	-	Ref.	0.68
Secondary	23.96	0.14	[-0.78-1.05]		32.9	1.09	[0.51-2.31]		25.50	0.67	[-0.29-1.63]		46.0	0.99	[0.58-1.70]		26.29	0.79	[-0.37-1.95]		60.3	1.36	[0.56-2.93]	
None or primary	23.48	-1.78	[-4.61-1.05]		42.9	0.68	[0.09-5.03]		24.69	-0.12	[-1.77-1.52]		42.4	0.90	[0.36-2.25]		26.68	1.00	[-0.41-2.42]		61.7	1.29	[0.56-2.93]	
**Socio-occupational groups**																								
Executives. mangers	23.19	-	Ref.	0.028	21.	-	Ref.	0.066	24.87	-	Ref.	0.67	44.3	-	Ref.	0.60	25.76	-	Ref.	0.27	51.9	-	Ref.	0.75
Never employed	22.28	-1.39	[-2.66--0.12]		9.2	0.35	[0.11-1.14]		23.49	-1.04	[-5.08-3.01]		25.0	0.57	[0.05-6.74]		19.41	-7.36	[-15.01-0.29]		0.0	unest.	unest.	
Tradespeople, shop and market sales people	23.52	-0.11	[-1.30-1.09]		50.0	0.97	[0.38-2.51]		25.10	0.75	[-0.38-1.88]		38.5	1.12	[0.60-2.09]		25.85	0.1	[-1.24-1.25]		58.7	1.06	[0.53-2.14]	
Intermediate occupation	26.69	3.29	[-0.05-6.64]		25.0	3.98	[0.35-45.17]		25.10	0.07	[-1.60-1.73]		44.3	0.72	[0.28-1.83]		26.83	0.84	[-0.51-2.20]		54.7	1.33	[0.62-2.85]	
Employees	24.09	0.32	[-0.97-1.62]		38.6	1.96	[0.72-5.34]		25.48	0.73	[-0.74-2.20]		52.3	1.63	[0.72-3.69]		26.08	0.40	[-116-1.97]		61.4	1.82	[0.73-4.53]	
Workers	24.35	-0.06	[-1.43-1.30]		40.0	1.21	[0.42-3.36]		25.11	0.20	[-1.21-1.61]		43.4	1.00	[0.46-2.18]		26.69	0.74	[-0.67-2.15]		64.0	1.78	[0.78-4.06]	
**Having had children**																								
Yes	24.79	-	Ref.	0.0003	46.6	-	Ref.	0.0001	25.52	-	Ref.	0.001	46.8	-	Ref.	0.087	26.16	-	Ref.	0.96	57.4	-	Ref.	0.69
No	23.07	-1.61	[-2.49--0.73]		20.0	0.25	[0.13-0.49]		24.16	-1.39	[-2.21--0.56]		38.8	0.67	[0.42-1.06]		26.05	-0.03	[-1.11-1.05]		55.0	0.88	[0.48-1.62]	
**Parental nationality**																								
Both French	23.13	-	Ref.	0.056	22.7	-	Ref.	0.037	25.10	-	Ref.	0.80	44.0	-	Ref.	0.38	26.11	-	Ref.	0.79	54.9	-	Ref.	0.39
Sub-Saharan African	22.97	-0.02	[-2.10-2.05]		9.1	0.40	[0.04-3.93]		24.75	-0.87	[-2.45-0.72]		34.2	0.52	[0.21-1.27]		24.33	-1.16	[-3.72-1.39]		36.4	0.52	[0.11-2.38]	
Middle Eastern/ North African	24.41	1.56	[0.44-2.68]		32.6	2.29	[0.97-5.41]		25.62	0.24	[-1.26-1.74]		44.7	0.85	[0.37-1.94]		26.93	-0.25	[-1.93-1.42]		69.4	1.23	[0.45-3.37]	
Asian	24.79	1.46	[-0.95-3.87]		55.6	5.00	[0.89-28.00]		24.93	-0.38	[-2.58-1.81]		53.3	1.31	[0.39-4.38]		22.78	-1.76	[-5.36-1.84]		20.0	0.37	[0.03-5.11]	
Western European/ North American	25.02	1.32	[-0.50-3.13]		42.9	2.05	[0.55-7.61]		25.89	0.40	[-1.29-2.09]		52.0	1.25	[0.50-3.15]		26.82	0.50	[-1.13-2.13]		66.7	1.57	[0.60-4.15]	
One French and one foreign parent	24.73	1.92	[0.28-3.55]		52.6	6.20	[1.87-20.61]		24.67	-0.71	[-2.87-1.44]		46.2	0.90	[0.27-2.97]		26.55	0.56	[-1.35-2.47]		76.5	3.05	[0.89-10.38]	
Other	23.49	1.30	[-1.51-4.11]		16.7	1.39	[0.12-15.70]		26.23	0.75	[-2.11-3.61]		75.0	3.74	[0.65-21.67]		25.97	0.80	[-2.23-3.82]		57.1	1.99	[0.35-11.35]	
**Proportion of life time spent in France**																								
Less than 50%	23.43	-	Ref.	0.044	23.3	-	Ref.	0.017	24.86	-	Ref.	0.16	41.0	-	Ref.	0.27	23.67	-	Ref.	0.0036	26.9	-	Ref.	0.0057
50% to 99%	23.40	0.70	[-0.63-2.02]		26.5	1.94	[0.65-5.76]		25.83	0.81	[-0.38-1.99]		51.9	1.55	[0.80-2.99]		26.53	3.00	[1.25-4.75]		63.3	6.00	[2.01-17.92]	
100%	23.53	1.36	[0.26-2.45]		27.5	3.57	[1.46-8.72]		24.98	-0.06	[-1.34-1.23]		42.9	1.07	[0.53-2.18]		26.19	2.76	[0.89-4.64]		56.3	5.03	[1.59-15.95]	

*Overweight prevalence (%).

^Ɨ^Consumption units.

*Unest.:* non computable.

In order to quantify the crude associations between overweight and independent variables, we have computed univariate multilevel regression models (see Table 2). Multivariate associations between BMI or overweight and independent variables were estimated and quantified by calculating coefficients (Coef.) or odds ratios (OR), and their 95% confidence intervals (95% CI) presented between brackets (respectively linear or logistic ones, using the “xtreg” and the “xtmelogit” command in Stata11 software, specifying that the collected data were clustered by census block). All the analyses were performed for men and women separately, since the literature usually reports gender differences for factors associated with overweight [21,22].

Finally, multivariate multilevel regression models were fitted with all the independent variables. Since interactions were found between age and socio-professional category, and between age and having a child or not in association with overweight (respectively p = 0.02 and 0.007), we fitted models in 3 age groups in both genders to assess in greater detail the role of parental nationality and the proportion of lifetime spent in France for different ages.

All the multilevel regression models were fitted on non weighted data. Because of possible redundancy between parental nationality and the proportion of lifetime spent in France, we also tested colinearity between these two variables by calculating the variance inflation factors (VIFs) in the regression models. Overall, most of the VIF values were under 3.0 (except for some slightly higher values, that never exceeded 5), which suggested that multicolinearity was not a serious problem [23]. The analyses were conducted using PASW Statistics 18 and Stata 11.

## Results

Our survey estimated that 36.7% (95%CI = [34.7-38.8]) of the adult, French-speaking population in the Paris metropolitan area was overweight in 2005: 32.7% (95%CI = [30.1-35.4]) of women and 41.2% (95% CI = [37.9-44.6]) of men (Table 1).

In univariate analysis, all the covariables taken into account in our analysis were significantly associated with overweight in both genders, with the exception of household income in men (Table 2). Interestingly, we observed significant differences in overweight prevalence according to parental nationality and proportion of lifetime spent in France in both genders. In women, the lowest overweight prevalence was in the Asian parentage category (23.2%) and in the both French parentage category (28.7%). On the contrary, those born to SSA or MENA parents had the highest prevalence of overweight (respectively 51.7%, OR = 1.96, 95%CI = [1.29-2.97], and 51.4%, OR = 2.17, 95%CI = [1.56-3.03]). Regarding lifetime spent in France, women overweight was the most prevalent in the “50% to 99%” category (39.8%, OR = 1.37, 95% CI = [0.99-1.90]). In men, overweight prevalence was even higher among the same category (51.2%, OR = 2.43, 95% CI = [1.63-3.61]) and remained higher among those who have lived 100% of their life in mainland France (40.1%) than those who have lived less than 50% (27.4%). Men of SSA parentage had an overweight prevalence below 30% (27.7%), while those born to one French and one foreign parent (56.4%), those of WENA parentage (55.5%) and those of MENA parentage (47.8%) had the highest prevalence.

The univariate analysis stratified by gender (Table 2) provided additional information. First, age was strongly associated with overweight for men and women (p < 0.0001), as well as having had children. A low household income was significantly associated with overweight in women but not in men, when a low education level and the socio-occupational group were associated with overweight in both genders. Regarding parental nationalities, with the reference category consisting of individuals born to two French parents, being of MENA or SSA parentage was associated, in women, with a higher risk of being overweight (respectively OR = 2.17, 95% CI = [1.56-3.03] and OR = 1.96, 95% C I= [1.29-2.97]. On the contrary, being of Asian parentage seemed to be a protective situation. In men, the highest risk of being overweight was among one French and one foreign parent category (OR = 2.01, 95% CI = [1.11-3.64]).

In Tables 3 and 4 are presented the different associations between parental nationality and proportion of the life spent in France and BMI as a continuous variable or overweight as a dichotomous one, among men and women and across different age groups, and adjusting for socioeconomic status.

First, regarding socio-economic status and demographics, we observed several differences between men and women, and also between age groups. Associations between BMI and income were significant only in the older age group. Among people ≥ 55 years of age, a lower income was associated with a higher BMI in women (Table 3) when the association was in an opposite way in men (Table 4). Second, in women, not having had any child was significantly associated with a lower BMI and a lower risk of being overweight in all age groups when, in men, both associations were significant in the youngest age group only (but the association with BMI was also significant in the 35–54 age group). In women, a lower educational level was also significantly associated with a higher BMI and a higher risk of being overweight in all age groups, but socio-occupational group was never so. On the contrary, among men, educational level was never associated with our outcomes.

Regarding parental nationality, significant associations were observed in women under 55 years of age (Table 3). In the two corresponding age groups, our adjusted models showed that women of MENA parentage were at higher risk for overweight than those of French parentage (OR = 2.00, 95% CI = [1.02-3.91] and OR = 2.04, 95% CI = [1.14-3.65] respectively). This parentage category had also a higher BMI among the second age group and was at higher risk of being overweight in the oldest age group (OR = 4.31, 95% CI = [1.06-17.49]). Women of SSA origin aged 35 to 54 years had also a significantly higher BMI (Coef. = 2.00, 95% CI = [0.49-3.50]) and a higher risk of being overweight (OR = 3.07, 95% CI = [1.42-6.67]). In men (Table 4), significant differences according to parental nationality were only observed in the youngest age group and for overweight. After the age of 35, their magnitude and their degree of significance decreased (for some of them, dramatically). The youngest men of MENA parentage had a significantly higher BMI (Coef. = 1.56, 95% CI = [0.44-2.68]) than those in the same age group born to two French parents. They had also more than twice the risk of being overweight but this was not significant (OR = 2.29, 95% CI = [0.97-5.41]). Young men born to one French and one foreign parent were at even greater risk of being overweight (OR = 6.20, 95% CI = [1.87-20.61]) and had also a higher BMI (Coef. = 1.92, 95% CI = [0.28-3.55]).

Regarding the proportion of lifetime spent in France, the only significant association with overweight in women was observed among the 35–54 years old group. Women who had spent between 50% and 99% of their lifetime in France were twice as high at risk of being overweight compared to those who had spent less than 50% of their lifetime in France (OR = 2.15, 95% CI = [1.22-3.76]). One last noteworthy result is that, in men, the risk of being overweight was highly correlated with having spent most of one’s life in France among youngest and oldest men: in the 18–34 years old group, OR = 3.57 (95%CI = [1.48-8.72]) for the “100%” category, and in men ≥ 55, OR = 6.00 (95% CI = [2.01-17.92] and OR = 5.03 (95%CI = [1.59-15.95]) for the “50% to 99%” and the “100%” categories, respectively. Those categories present also significantly higher BMIs.

## Discussion

Our data and results concerning the differences in overweight according to parental origin are the first ones to be published in France from a representative, population-based, health survey. Indeed, at best, only the respondent’s or case’s citizenship is available in French surveys or health information systems (and it is rarely, if ever, studied in epidemiological surveys on obesity or overweight). Until now, this has always precluded origin-based statistical portraits of the French population in the fields of public health and epidemiology in general. When almost a quarter of French people is estimated to have at least one immigrant parent or grandparent [24], it is more and more useful to identify this population as a group of interest. In this context, the aim of this research was to explore the impact of geographical and cultural origin and the proportion of one’s life spent in France on overweight across both genders and different age groups.

Since the socioeconomic factors of overweight have been analyzed and explained more thoroughly in many other studies, in France and in elsewhere [25,26], we will simply point out that the role of such factors differed according to gender and age groups in our study population. Indeed, a low household income or a low educational level were significant factors associated with overweight and a higher BMI in older women only while, in men, SES associations were not significant and more ambiguous. Actually, the relationship between socioeconomic characteristics and overweight is complex and would require further research to better understand the pathway through which they affect weight status. On the contrary, we found an overall association with the fact of having children among both men and women.

More importantly, we showed that, even after adjustment for SES, migration origin (as approximated by parental nationality) and the proportion of lifetime spent in France were associated with BMI and overweight, and that these associations differ according to gender and age groups. In women, a parental origin in the Middle East or North Africa was associated with a higher risk of being overweight (especially before the age of 55) and a higher BMI (between 35 and 54 years of age), and so were women of SSA parental origin in the middle age category. Only in the youngest men (< 35 years of age) did we observe any association with parental nationality, with a higher risk of being overweight when having a mixed nationality parentage (i.e., having been born to one French and one foreign parent) and a higher BMI when having a MENA parentage. Regarding the association between the proportion of lifetime spent in France and overweight, we observed that, in women, a proportion of 50% to 99% appeared to be associated with overweight, especially after the age of 35. In men, having spent more than half of one’s lifetime in France was associated with a higher risk of overweight but was particularly significant among oldest men.

Such results are consistent with the existing literature on migration and weight status (whether in immigrants or their descendants), which indicates that the length of residency in a host country has an impact on weight status. Since 2000, there have been numerous studies on this subject [27], mainly in the United States [28,29], but also in other countries [30,31]. Our results are not consistent with a previous French study [13], which found no association between the proportion of lifetime spent in France and overweight. We cannot fully explain this difference because the proportion data are not presented in that paper, but it may be due to the specific design and the data aggregation. Our results underscore the importance of including the duration of residency as an exposure measure in studies on immigration and weight.

These results have certain limitations. First, because of the study’s cross-sectional design, we cannot conclude that a higher proportion of lifetime spent in France caused overweight in this population, for we did not know the respondents’ weight status before they came to France or the chronological direction of the association. The next waves of the SIRS cohort will enable us to collect BMI data prospectively and to obtain retrospective data at earlier ages, which may shed light on the pathway through which geographical origins impact BMI. Second, the accuracy of self-reported weight and height can be a problem, even if, in the SIRS survey, this problem was avoided somewhat by the fact that the interviews were conducted face-to-face. A previous French study showed that such self-reporting can lead to significant underestimations of BMI (0.29 and 0.44 kg/m^2^ for men and women, respectively) [32]. This would have biased our results if these underestimations were specific to some of the population groups, as pointed out in previous studies [33,34]. However, considering the strength of the estimated associations, we think it rather doubtful that such bias could account for them completely. We have to mention also the issue linked to our sample size: the number of observed subjects in some categories is rather low (especially when analyzing different gender and age subgroups), which reduces the statistical power of our results. Also, our sample being representative of the Paris metropolitan area, our results and analyses must be considered in this particular context and cannot be extrapolated to the situation of the whole population in France. Certain important factors were not asked about in this survey (e.g., dietary practices and lifetime weight histories, but also measures of - or proxies for - the acculturation process). Obviously, this lack of information limits the interpretation of our results. For instance, we did not have any details about individuals’ migration history, with the result that we did not know exactly where they had spent that part of their life outside France or during what period(s) of their life they had been abroad.

Without ignoring the fact that genetic factors could explain some of the differences observed, we will focus the following discussion on the cultural, historical and economic dimensions of our results, since data on genetic characteristics were not available in our dataset.

Lifetime proportion spent in a given country and a given host culture can modulate the influence of a person’s geographical family or personal origin. It is usually used in migration studies of the health impact of exposure to a given lifestyle, but with different thresholds. For example, in France, a recent study on migration biographies differentiated between individuals who had spent at least one year outside France (migrants) and those who had not [10]. We think that such a threshold may have some limitations and leads to a broad definition of migrant. In this paper indeed, 50% of the respondents were immigrants (foreigners born abroad), but 24% were French people with no migratory descent [10]. In our study, we attempted to identify a threshold of lifetime proportion spent in France that was meaningful for people in terms of socialization and cultural exposure. We chose to isolate those who had spent their entire life in France because of their distinctiveness, and we used a threshold of 50% of lifetime to differentiate the others (< 50% vs. ≥ 50%). Having spent 50% or more of one’s life in a country may have a particular impact in terms of socialization, acculturation, and (or through) exposure to the host lifestyle (regardless of when these periods of time were situated in the individual’s life and even their duration).

Cultural issues - specifically tastes and dietary habits and preferences [14,35], but also esthetic norms [36-38] - are often cited to explain the association observed between origin and weight. For instance, a French study has shown how these factors can be a source of conflict between Moroccan mothers and their daughters born and raised in France, whose norms and dietary habits these mothers may find irrelevant and culturally illegitimate [39]. Socioeconomic factors are the other main hypothesis for explaining such differences, since the socioeconomic status of immigrants (or of people born to immigrants) is generally lower than that of native-born people (or of individuals born to native-born people). We will be cautious in any cultural interpretation of our results because no detailed data on these aspects were available in our dataset to further explore them and because our results are based on broad, constructed origin categories, each encompassing a wide diversity of cultures. Nonetheless, we should point out that, since all our regression models were adjusted for income, socio-occupational status and education level, we controlled for these socioeconomic aspects.

In the literature, acculturation is often cited as a potential explanation for the association between geographical origin and duration of residency in the host country and BMI. Acculturation is the transformation that groups and individuals undergo when they come in contact with another culture. It has been suggested that the degree to which immigrants acculturate to the majority culture after they arrive in the host country influences BMI [28,40], since nutrition and diet – as well as a sedentary lifestyle with less regular exercise [41] – may be changing behaviours, adopted by immigrants in the receiving country. This is observed frequently in the United States as the result of exposure to the American environment and the adoption of American dietary and physical activity patterns [42,43]. Our results (especially the fact that the proportion of lifetime spent in France was associated with overweight) may provide a clue to a similar effect regarding exposure to French dietary and physical activity patterns, which are increasingly obesogenic [1,41], albeit not to the same level as in other developed countries. Other French studies mention nutrition transition, which occurs when “traditional” dietary patterns are gradually replaced with “modern” ones. This change seems to particularly affect French people of foreign parentage [14,41], i.e., those who account for a substantial portion of the “50% to 99%” group. Indeed, the French diet has been identified as distinct, with less fat-reduced food [44] and more fat overall and more saturated fat than the American diet, for instance [45]. However, in a country comparatively less affected by the modern obesity epidemic than many other developed or emerging countries, the French environment also contains aesthetic norms that still favour slenderness very strongly, as shown by authors who compared France and the United States in this regard [46]. These norms place more pressure on women than on men [47], which could explain why having spent one’s entire life in France was, in women of French as well as African origin, strongly associated with a lower risk of overweight. Finally, mention should be made here of the specific case of people born to one French and one foreign parent, who, in the case of men, had the highest risk of overweight in multivariate analysis. This finding may indicate that the combination of a French environment and a mixed food culture has a distinct impact on the risk of overweight, possibly through the adoption of especially negative or conflicting food habits.

## Conclusions

These results are informative with regard to the potential cultural determinants of overweight in the specific French context. They provide an additional clue to the association between migration profile and weight status observed in several studies in other countries. Since globalization has modified international and transcultural influxes of people, it is a challenge for public health authorities to better understand the impact of migration descent and the lifestyle of the host country on health. Our work has highlighted a potential negative impact of exposure to the French way of life. It has also identified several useful points for further study and for better understanding the association between overweight and geographical origin. Of course, to perform a more thorough statistical analysis of the relationships between migration history and overweight, more-detailed (preferably longitudinal) data would be needed, such as on migration and weight, and family and personal biographies, but also on socialization and social integration in the host country, the persistence of old cultural norms, attitudes and behaviours, and the acquisition of new ones, to identify and analyze the acculturation process and the lifestyle impact on overweight. Nevertheless, our results show that the proportion of lifetime spent in France and parental nationality are two simple, easy-to-collect data that could be relevant indicators for describing, comparing and monitoring overweight prevalence according to people’s origins in mixed populations.

## Competing interest

The authors declare that they have no competing interests.

## Authors’ contributions

JMF: has made substantial contributions to conception and design, acquisition of data, analysis and interpretation of data and has been involved in drafting the manuscript and revising it critically for important intellectual content. FG: has made substantial contributions to conception and design, or acquisition of data, analysis and interpretation of data and has been involved in drafting the manuscript and revising it critically for important intellectual content. CT: has been involved in drafting the manuscript and revising it critically for important intellectual content. IP: has been involved in drafting the manuscript and revising it critically for important intellectual content. PC: has made substantial contributions to conception and design, or acquisition of data, analysis and interpretation of data and have been involved in drafting the manuscript and revising it critically for important intellectual content. All authors have given final approval of the version to be published.

## Pre-publication history

The pre-publication history for this paper can be accessed here:

http://www.biomedcentral.com/1471-2458/12/937/prepub
